# Weight Bearing Asymmetry and Functional Ambulation Performance in Stroke Survivors

**DOI:** 10.5539/gjhs.v4n2p87

**Published:** 2012-03-01

**Authors:** B. O. A. Adegoke, O. Olaniyi, C. O. Akosile

**Affiliations:** Physiotherapy Department College of Medicine, University of Ibadan Ibadan, Nigeria; Physiotherapy Department, Federal Medical Centre Owerri, Nigeria; Medical Rehabilitation Department, College of Health Sciences Nnamdi Azikiwe University, Nnewi Campus Awka, Anambra State, Nigeria

**Keywords:** Stroke survivors, Weight Bearing Asymmetry, Functional ambulation, Emory Functional Ambulation Profile

## Abstract

This study evaluated asymmetry of weight bearing on the lower limbs and the association between percentage weight bearing asymmetry (PWBA) and functional ambulation performance in ambulant stroke survivors. Participants were 53 stroke survivors (male = 35, female = 18) aged 40-86 years (mean=58.87; SD=9.21years) with hemiparesis. Weight bearing through the lower limbs in standing was assessed by two juxtaposed bathroom weighing scales while functional ambulation performance was evaluated with the Emory Functional Ambulation Profile (E-FAP). Data were summarized with mean and standard deviation and further analyzed using the Pearson product moment correlation at 0.05 alpha level. Participants bore 60.3% (SD =7.1%) of their body weights on the unaffected legs and had a mean PWBA of 20.8 % (SD=14.7%). There was a significant positive correlation (r = 0.675, p < 0.0001) between PWBA and total E-FAP scores of participants. PWBA could hence be used to monitor functional ambulation recovery in stroke survivors.

## 1. Introduction

Stroke is the third leading cause of morbidity and a major cause of long-term disability in the elderly (Bath, 2005). A high percentage of stroke survivors often experience long-term balance and mobility problems that may result in falls during rehabilitation or even after discharge from treatment (Falconer *et al.*, 1994; Weedesteyn *et al.*, 2008). Further, individuals with hemiparesis as a result of stroke often have difficulty accepting and bearing weight on the paretic leg hence they commonly exhibit asymmetry in standing and during ambulation with greater proportion of the body weight being borne on the non-paretic leg (Mercer, 2003). Distribution of body weight appears to be a significant factor influencing postural stability and one of the measures of balance used to quantify the extent of deficiency in postural control in stroke survivors (Nichols, 1997). Walking dysfunction is the most commonly reported limitation after stroke (Bohannon *et al.*, 1988) and can markedly affect independence, quality of life, and participation (Schmid *et al.*, 2007).

There is no consensus as to the percentage of the body weight which is borne on the affected leg in stroke survivors though it has been generally reported that the unaffected leg bears a disproportionately greater proportion of the weight than the affected leg; a phenomenon known as Weight Bearing Asymmetry (WBA). The percentage of the body weight that is borne by the unaffected leg of the stroke survivor has hence variously been put at 61% (Sackley *et al.*, 1992), 63% (Caldwell *et al.*, 1986), and 70% (Shumway-Cook *et al.*, 1998, Mizrah *et al.*, 1989). Sacley *et al*. (1992) further submitted that the unaffected leg may bear as much as 90% in severely affected individuals. Percentage WBA was found by Nichols (1997), to be up to 27% while Sorinola (2000), reported the percentage WBA in a similar study to be between 23.6+15.0% and 21.0+8.4%.

Functional ambulation is the ability of a person to walk with maximal independence while spending the shortest time under various environmental circumstances. Although there is no established or gold criterion specifically for the assessment of ambulation, there are tests for gait speed (Wolf, *et al.*, 1999). Gait speed has hence been used to evaluate stroke disability and walking recovery, estimate future health status and function, and predict level of community ambulation in stroke patients (Wade *et al.*, 1992; Lord *et al.*, 2004; Schmid, *et al*. 2007) and as the primary outcome measure of rehabilitation in stroke patients (Wade *et al.*, 1992). Goldie *et al*. (1996) reported that walking ability is markedly compromised following stroke and that majority of stroke survivors who regain their walking ability fail to achieve a normal walking speed of 1.2metres per second; the speed demonstrated by healthy adults. Ambulation performance has been assessed by the timed 10-metre walk test by various authors (Miller *et al.*, 2002; Dean *et al.*, 2000; Wolf *et al.*, 1999). However, though, the timed 10-metre walk test is a valid test of mobility, it does not typically test the ability to move round obstacles and over different terrains (Wolf *et al.*, 1999). It thus has limited applicability since an assessment of ambulation should include environmental variables encountered in daily living such as different terrains, obstacles and stairs (Robinett and Vondrain, 1988). An example of a clinically based measure is the Functional Ambulation Profile (FAP), which is a timed walking test specifically designed to track the progress of patients with neurological impairment (Perry *et al.*, 1995).

Previous studies have investigated the correlations between WBA, gender, motor function and period since onset of stroke (Adegoke and Akinkoye, 2003), WBA and rate of fall (Cheng *et al.*, 2001), WBA, functional ability and selected gait parameters (Adebisi, 2003) and WBA, motor function recovery and functional ability (Sorinola, 2000). The influence of WBA on stroke survivors’ ability to negotiate obstacles and move over different terrains (functional ambulation performance) has not received much attention in literature. This is important in a country like Nigeria where many communities are unpaved and trekking is the most common mode of transportation in the rural areas and among the poor. This study therefore investigated WBA in a group of hemiparetic stroke survivors and the relationship between Percentage Weight Bearing Asymmetry (PWBA) and functional ambulation performance assessed using the Emory Functional Ambulation Profile (E-FAP)

## 2. Research Methods

### 2.1 Subjects

Fifty three consecutive patients (35 males and 18 females) of ages 40-86 years who had hemiparesis post-cerebrovascular disease (CVD) and were receiving physiotherapy treatment at the University College Hospital (UCH), Ibadan, Ring Road State Hospital, Ibadan, Lagos University Teaching Hospital (LUTH) and Lagos State University Teaching Hospital (LASUTH), Lagos at the time of data collection participated in this ex-post facto study. All participants met the following inclusion criteria:

1. No visual or hearing problem.
2. No other neurological problem apart from stroke.
3. No orthopaedic problems of the lower limbs that may affect lower limb function.
4. Able to understand (comprehend) and follow instructions.

### 2.2 Instruments

Instruments for data collection comprised two bathroom weighing scales (Hana, Germany) and the Emory Functional Ambulation Profile (E-FAP). The E-FAP has been reported to have high construct and concurrent validity, inter-rater reliability ≥ 0.997 and test-retest reliability≥0.998 for the five sub-tasks.

### 2.3 Procedure

The study was subject to the approval of the University of Ibadan/University College Hospital Institutional Review Committee on human subject research. Subjects also gave their informed consent while the managements of the hospitals where the data were collected permitted the conduct of the study.

Age and sex of each subject were recorded. Two bathroom weighing scales were then checked for accuracy against standard metal weights to ensure that the position of a load on the scale had no effect on the reading obtained as suggested by Caldwell *et al*. (1986). Each subject was then requested to stand on one of the weighing scales to measure the total body weight in kilograms to the nearest kilogram. The two scales were used alternately for total weight measurement to avoid overusing either of them. The two weighing scales were then juxtaposed at 10cm distance apart and at a distance of 2 meters from the wall (Caldwell *et al*. 1986) and the subjects instructed to stand as erect as possible while placing one foot on each scale and looking forward. The weight on each scale was read off and recorded. Three trials were conducted for each subject with the subject stepping off the scales between trials to allow the scales return to zero. The mean of the three readings of each weighing scale were calculated for each limb and taken as the weight borne on that lower limb. Percentage weight bearing asymmetry (PWBA) was then calculated according to the following formula:

PWBA = [(Weight on the unaffected leg – weight on the affected leg)/Total Body Weight] × 100 (Nichols, 1999).

Subjects were then timed as they performed the five tasks of E-FAP namely: 5-metre walk on hard surface, 5-metre walk on carpeted surface, up- and- go, obstacle negotiation, and ascending/descending stairs. The time taken to complete each task was recorded as the score for each task while the total time taken to complete all the tasks represented the total profile (E-FAP) score (Wolf, *et al.*, 1999). All E-FAP evaluations were carried out by one of the researchers (OO) to eliminate inter-rater variations.

### 2.4 Data Analysis

Data were analysed using descriptive statistics of mean, standard deviation and percentages while Pearson product moment correlation method was used to investigate associations among PWBA, task scores and total E-FAB score at 0.05 alpha level.

## 3. Analysis Result

The subjects’ parameters and comparison between the parameters of male and female subjects are presented in Table 1. Subjects’ mean age and body weight were 58.87 ± 9.21 years and 70.51 + 11.0 kg respectively. Percentage weight bearing on the unaffected leg was 60.3 ± 7.1% (range= 50.9 - 78.6%) and all subjects bore more weight on their unaffected leg. The mean PWBA of the subjects was 20.8 + 14.7% (range= 1.4 - 57.1%) and the mean time since stroke was 13.58 ± 14.13 months (range = 1- 60 months). Mean PWBA for first, second and third trials were 20.86 ±14.82, 20.72 ±14.56, and 20.91±14.72 respectively with corresponding percentage coefficient of variations (%CV) of 71.04%, 70.3%, and 70.4% for the respective trials. Male and female subjects did not differ significantly on any of the parameters. The total E-FAP and five subtask scores of the subjects are presented in Table 2. The mean total E-FAP score of the subjects was 91.82 + 41.45 seconds (range = 44.8-22.31 seconds). The lowest mean score (best performance) of 10.40 + 6.87 seconds was recorded for the 5-metre walk on hard surfaced floor task while the highest mean score (worst performance) of 32.95 + 11.99 seconds was recorded with the obstacle negotiating task.

**Table 1:** Characteristics of subjects

Variable	All subjects (n = 53) X ± S.D	Male subjects (n = 34) X ± S.D	Female subjects (n = 19) X ± S.D	95% CI	p
Age (years)	58.87± 9.21	59.56± 9.45	57.63± 8.87	-3.39 to 7.24	0.470
Height (meters)	1.66± 0.08	1.65± 0.08	1.67± 0.09	- 0.07 to 0. 03	0.487
Weight (kg)	70.51± 11.01	71.00± 11.61	69.93± 10.09	-5.01 to 7.75	0.669
BMI (kg/m2)	25.58± 3.66	25.91± 3.77	24.98± 3.47	-1.18 to 3.04	0.379
TSS (months)	13.58± 14.13	15.97± 15.86	9.32± 9.25	-1.33 to 14.64	0.100
PWA	39.60 ± 7.36	38.74± 7.81	41.72± 6.36	-6.60 to 1.84	0.262
PWU	60.40± 7.36	61.26± 7.81	58.88± 6.36	-1.84 to 6.60	0.262
PWBA	20.85± 14.67	22.59± 15.56	17.75± 12.73	-3.57 to 13.25	0.253

NB:

TSS = Time since stroke

BMI = Body mass index

PWA = Percentage weight on affected leg

PWU = Percentage weight on unaffected leg

PWBA =Percentage weight bearing asymmetry

**Table 2:** Emory Functional Ambulation Profile (E-FAP) scores of subjects

Variable	Range	X ± S.D
5m-HF (seconds)	3.82 – 34.0	10.4± 6.9
5m-CF (seconds)	3.95 – 35.76	11.6± 7.9
UGT (seconds)	6.72 – 60.44	20.0± 11.5
ONT (seconds)	13.12 – 58.90	32.4± 12.1
ADS (seconds)	6.90 – 42.87	16.7± 8.7
E-FAP (seconds)	44.8 – 223.1	91.8 ±41.5

NB:

5m-HF=5-metre walk on hard surfaced floor

5m-CF=5-metre walk on carpeted (rugged) floor

UGT= Up-and-go task

ONT=Obstacle negotiating task

ADS=Task of ascending and descending stairs

E – FAP =Emory Functional Ambulation Profile

Correlations among PWBA, total E-FAP score, and subtask scores are presented in Table 3. There were significant positive correlations between PWBA and the total E-FAP score (r = 0.675, p < 0.0001) as well as between PWBA and each of the E-FAB subtask scores (r ≥ 0.509, p< 0.0001). The correlation (r = 0.749) between PWBA and the total E-FAP score was higher than the correlations between PWBA and the subtasks’ scores (r ≥ 0.561). The highest correlation (r= 0.680) was obtained between PWBA and the “up-and-go” subtask while the least correlation (r=0.509) was between E-FAB and the “obstacle negotiation” subtask. There were also significant positive correlations between the scores on the different subtasks; the highest correlation (r= 0.961) occurring between scores on the “5metre-walk on the carpeted floor” and scores on “up-and-go” task. The least correlation (r= 0.528) was obtained between scores on both “5-meter walk on the hard floor” and “5meter- walk on carpeted floor” and scores on “obstacle negotiating” task. There were also indirect but not significant correlations between time since stroke and both PWBA and E-FAP score.

**Table 3:** Correlation matrix for all variables

	Ht	Wt	WBL	WBR	5m-HF	5m-CF	UP&GO	TSS	NO	ADS	E-FAP	PWBA
Height	1.000												
Weight	0.451**	1.000											
WBL	0.298*	0.591**	1.000										
WBR	0.332*	0.761**	0.058	1.000									
5m-HF	-0.090	-0.128	-0.470**	0.354**	1.000								
5m-CF	-0.112	-0.145	-0.450**	0.307**	0.919**	1.000							
UP&GO	-0.065	-0.097	-0.506**	0.382**	0.961**	0.865	1.000						
TSS	-0.022	0.171	-0.195	0.020	-0.211	-0.159	-0.175	1.000					
NO	-0.029	0.109	-0.251	0.481**	0.528**	0.528**	0.587**	0.066	1.000				
ADS	-0.027	-0.011	-0.336	0.386**	0.830**	0.790**	0.833**	-0.249	0.568**	1.000			
E-FAP	-0.083	-0.064	-0.458	0.432**	0.945**	0.888**	0.951**	-0.161	0.747**	0.895**	1.000		
PWBA	-0.039	-0.035	-0.777	0.569**	0.651**	0.601**	0.680**	-0.166	0.509**	0.538**	0.675**	1.000	

WBL= Weight borne on left lower limb, WBR= weight borne on right lower limb.

5m-HF= 5 metre walk on hard floor, 5m-CF= 5 metre walk on carpeted floor

UP& GO= Up and go task, TSS= Time since stroke, NO= Negotiating obstacle

ADS= Ascending and descending stairs, E-FAP= Overall E-FAP score

PWBA= Percentage weight bearing asymmetry

* =Significant correlation at 0.05 level (2-tailed)

** = Significant correlation at 0.01 level (2-tailed)

## 4. Discussion

This study investigated weight bearing asymmetry in standing in a cohort of ambulant hemiparetic stroke survivors and the correlation between percentage weight bearing asymmetry and functional ambulation performance. Functional ambulation was assessed using the Emory Functional Ambulation Profile (E-FAP). All the subjects (100%) in this study bore more weight on their unaffected leg. This percentage is higher than the 93% reported by Adegoke and Akinkoye (2003) and the 80% by Bohannon and Larking (1985) and Caldwell *et al*. (1986). However, subjects in this study bore a mean of 60.3% of their body weight on their unaffected leg which is similar to the 63.4% and 61.0% reported by Caldwell *et al*. (1986) and Sackley *et al*. (1992) respectively, but lower than the 70.0% reported by Shumway-Cook *et al*. (1988) and Mizrahi *et al*. (1989) and higher than the 57.6% reported by Adegoke and Akinkoye (2003). Guillebastre *et al*. (2011) opined that the probability of a stroke survivor walking without a cane is less than 5% when the paretic lower limb is not loaded more than 40%. This implies that an average participant in this study may not require a cane. The mean PWBA of 20.81% observed in this study is similar to the 23.64% and 21.02% reported for the two groups of hemiparetic subjects in the work of Sorinola (2000). The differences in the values of PWBA reported by the different studies might be a reflection of the severity of the stroke (Sackley, 1990) and/or how soon after the onset of the hemiplegia the subjects were evaluated for asymmetry. Expectedly, as submitted by Caldwell *et al.*, (1986), long-standing cases would have received variable amounts of treatment that might include instruction on weight transfer through the affected leg and a consequent possibility of reacquiring some degree of ability to redistribute weight on the hemi-paretic leg. Subjects in this study have suffered stroke for 1-60 months and must have been receiving varying amount and quality of physiotherapy before taking part in this study though the amount of physiotherapy which subjects have received before taking part in this study was not considered as a variable. However, although measures of gait symmetry (swing time, stance time and step length) have been reported to demonstrate a systematic linear trend to greater asymmetry in the later stages poststroke, velocity, neurological deficit and lower extremity motor impairment did not (Patterson *et al.*, 2008).

Slight variations were observed between the mean PWBA obtained during the three trials. Considering the stability of the scores obtained for the three trials, it may not be necessary to subject patients to such trials in clinical settings. However, the coefficients of variation obtained suggest considerable variations among participants that may be reflective of their stages of recovery. The PWBA and %CV were lowest for the second trial and highest for the third trial thus suggesting that two trials may be optimum. It is plausible that participants were learning during the first trial while some degree of fatigue might have set in during the third trial.

The mean total E-FAP score of 91.82 seconds observed in our subjects was lower than the 103.51 seconds reported by Baer and Wolf (2001). As with weight-bearing asymmetry, we suspect that the severity of the initial stroke and how soon after the stroke the subjects were evaluated for functional performance could have been responsible for the observed differences. The lower the E- FAP score, the better the functional performance of stroke survivors. There was a statistically significant positive correlation between PWBA and E-FAP scores which implies that as the PWBA decreases, subjects improve in their functional ambulation performance and as a result, the time taken by subjects to complete ambulatory tasks (E.FAP score) reduces. Sackley *et al*. (1990) had earlier submitted that the observed weight bearing asymmetry (WBA) in hemparetic stroke subjects correlated significantly with motor function and functional activities of daily living while Sackley *et al*. (1992) concluded that WBA is linked with impaired motor function and in turn with decreased functional ability in stroke patients. Winstein (1989) had similarly observed that WBA was positively correlated with walking ability.

There were significantly positive correlations between the PWBA and participants’ mean total EFAP and subtask EFAP scores. The correlations were weakest between PWBA and scores on the obstacle (r= 0.561) and stairs (r = 0.625) subtasks which are subtasks that involve activities that are more challenging than level walking. Cromwell and Wellmon (2001), Nadeau *et al*. (2003) and Troy and Grabiner (2005) had variously shown the increased challenge of stairs’ ascent and descent compared to level walking and that the obstacle task requires quick restoration of dynamic balance. To maintain balance in the two tasks, the direction of body segments changes while kinematics and kinetics including weight bearing asymmetry are considerably higher than for level walking (Cromwell and Wellmon, 2001). Changes in the E-FAP scores may hence not be expected to have as much effect on performances on the obstacle and stair subtasks as on the other subtasks. This submission is supported by the findings from this study that the obstacle subtask score correlates only strongly with the total EFAP score and just fairly with other subtasks. It appears that poststroke individuals require bearing more weight on their unaffected limbs during the obstacle and stairs subtasks than they would during the performance of the other EFAP subtasks. We therefore suggest that exercises or treatment approaches aimed at influencing dynamic balance restoration should be included in poststroke management to facilitate better gait in the performance of the stairs and more importantly the obstacle subtasks. This position is supported by Hesse *et al*. (1995) and Akosile (2008). This recommendation will be in tandem with the suggestion by Genthon *et al*. (2008) that weight bearing asymmetry may not be the principle target of rehabilitation programs aimed at restoring standing balance after stroke since weight bearing asymmetry of standing stroke patients is not the primary cause of postural imbalance but a consequence of impaired control of postural stabilization involving both limbs.

We recommend that future studies evaluating therapy effectiveness in stroke using the EFAP should also use PWBA as an outcome measure and correlate this with EFAP scores at both pretest and posttest. This may indicate the extent to which our conclusions are justifiable.

### 4.1 Clinical implication

This study has shown that as the PWBA decreases, the E-FAP scores of subjects decrease; a low E-FAP score being an indication of a high functional ambulation performance. The PWBA can be used to monitor the response of post stroke hemiplegic patients to rehabilitation programs generally and functional ambulation performance specifically. The current practice whereby physiotherapists set the goal of achieving symmetrical weight distribution in their stroke patients is hence justified. The evaluation of PWBA does not require any sophisticated equipment and can be accommodated by the schedule of a busy physiotherapy clinician.
